# Mitochondrial DNA in Exercise-Mediated Innate Immune Responses

**DOI:** 10.3390/ijms26073069

**Published:** 2025-03-27

**Authors:** Xin Wen, Jingcheng Fan, Xuemei Duan, Xinyi Zhu, Jianzheng Bai, Tan Zhang

**Affiliations:** School of Exercise and Health, Shanghai University of Sport, Shanghai 200438, China

**Keywords:** mtDNA, innate immune, exercise, inflammation

## Abstract

Mitochondria are considered as “the plant of power” with cells for a long time. However, recent researches suggest that mitochondria also take part in innate immune response to a great extent. Remarkably, mtDNA was reported to have immunnostimulatory potential in 2004. Since then, there has been rapid growth in understanding the role of mtDNA in innate immune. The mtDNA is released into cytosol, extracellular environment, or circulating blood through BAK/BAX pore, mPTP, and GSDMD pore upon mitochondrial damage, where it is recognized by PRRs including TLR9, cGAS, and NLRP3, thereby triggering innate immune response. On the other hand, regular exercise has been recognized as an effective intervention strategy for innate immune response. Some studies show that chronic moderate-intensity endurance exercise, resistance training, HIIT, and moderate-intensity acute exercise enhance mitochondrial function by promoting mtDNA transcription and replication, thus blunting the abnormal release of mtDNA and excessive innate immune response. On the contrary, high-intensity acute exercise elicits the opposite effect. Nevertheless, only a very small body of research by far has been performed to illustrate the impact of exercise on mtDNA-driven innate immune response, and an overall review is lacking. In light of these, we summarize the current knowledge on the mechanism mediating the release of mtDNA, the role of mtDNA in innate immune response and the influence of exercise on mtDNA leakage, hoping to pave the way to investigate new diagnostic and therapeutic approaches for immunopathies.

## 1. Introduction

The innate immune system is the first defense line against the invasion of pathogens, innate immune cells recognize pathogen-associated molecular patterns (PAMPs) or damage-associated molecular patterns (DAMPs) through their pattern recognition receptors (PRRs), thereby activating distinct signaling pathways to produce inflammatory cytokines and interferon which ultimately trigger the innate immune responses [1]. Mitochondria are considered as “the plant of power” with cells and have its unique DNA. The mitochondrial DNA (mtDNA) is a small, double-stranded circular molecule (16,569 bp) that encodes 13 oxidative phosphorylation mRNAs as well as 22 tRNAs and 2 ribosomal RNAs [2]. The mitochondria-binding protein mitochondrial Transcription Factor A (TFAM) which is originally identified as a transcriptional activator for mtDNA promoters, promotes mtDNA packing. However, mtDNA is prone to damage by reactive oxygen species (ROS) due to the lack of histone packaging and ineffective DNA repair mechanisms, resulting in the formation of oxidized mtDNA (ox-mtDNA) [3]. Significantly, mtDNA was reported to have immunostimulatory potential in 2004 [4]. In the past two decades, numerous studies have reinforced that the mtDNA released into cytosol, extracellular environment or circulating blood under pathologic conditions, act as a DAMP, can be recognized by immune receptors such as toll-like receptor-9 (TLR9), cyclic GMP-AMP synthase (cGAS) or nucleotide-binding oligomerization domain-like receptor family, pyrin domain-containing 3 (NLRP3), and then boost multiple signaling pathways to trigger innate immune response. However, the potential role and underlying mechanism of mtDNA in exercise-mediated innate immune response have been rarely reported, and an overall review is lacking. Based on these, this paper comprehensively summarizes the crucial signaling pathways by which mtDNA regulates innate immune response, and focuses on the latest research progress of exercise and mtDNA.

## 2. Mechanisms of mtDNA Release

**Figure 1:**
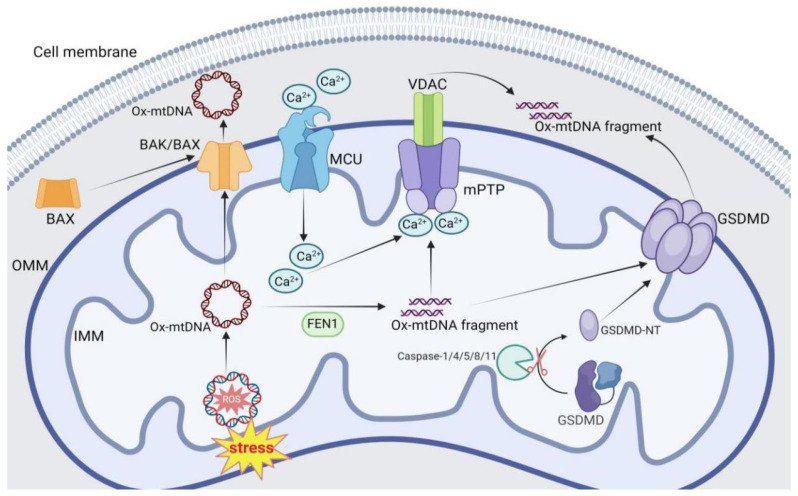
Mechanisms of mtDNA release. MtDNA can be released from mitochondria into cytosol, extracellular environment, or circulating blood through distinct pores, including BAK/BAX pore, mPTP, and GSDMD pore. Upon apoptotic and other stimulations, the activated BAX is transferred from cytosol to OMM and forms the BAX/BAK pore with BAK, releasing intact ox-mtDNA into cytosol. In addition, Ca^2+^ enters mitochondria via MCU in response to non-apoptotic stresses such as radiation, toxic exposure, viral infection, and gene mutation, thereby inducing mPTP opening and VDAC oligomerization. At the same time, the intact ox-mtDNA that formed within the mitochondria is cut by FEN1 into short fragments of 500–650 bp which are released through mPTP. In addition, the GSDMD is cut by Caspase-1/4/5/8/11 to form the GSDMD-NT fragments which are then inserted into the mitochondrial membrane to form the GSDMD pore, facilitating the release of mtDNA fragments or ox-mtDNA fragments.

The mtDNA is located in the mitochondrial matrix under physiological conditions. ROS is the product of oxidative phosphorylation, and remains low normally but increases sharply in response to stimulus such as radiation, hypoxia, oxidative stress. Subsequently, mtDNA is probably oxidized by the accumulated ROS as mentioned above [5,6]. The resulting ox-mtDNA then escapes from mitochondria through Bcl-2-associated K protein (BAK)/Bcl-2-associated X protein (BAX) pore, mitochondrial permeability transition pore (mPTP), or Gasdermin D (GSDMD) pore to trigger innate immune responses (Figure 1).

### 2.1. BAK/BAX Pore

The BCL2 family proteins, including pro-apoptotic proteins, pro-survival proteins and pro-apoptotic effector proteins, play a vital role in the regulation of apoptosis. The pro-apoptotic effector proteins BAK and BAX exist in the mitochondrial outer membrane (OMM) and cytoplasm in inactive form under normal physiological conditions, and the pro-survival members of the Bcl-2 family inhibit the activity of BAK and BAX. On the contrary, the activity of pro-survival members of Bcl-2 family is blocked, and the pro-apoptotic proteins are activated which lead to the activation and conformational changes of BAX, the active BAX then transfers from cytoplasm to OMM where it forms pores with BAK, resulting in the release of cytochrome c into cytosol upon apoptotic stimulations. Furthermore, only a small amount of BAX is needed for OMM permeability, and most of BAX is recruited from the cytoplasm after the release of cytochrome c to form pores with BAK [6,7].

Intriguingly, in addition to the release of cytochrome c during mitochondrial apoptosis, the BAK/BAX pore is also shown to be responsible for the release of mtDNA induced by caspase-independent cell apoptosis, thereby activating cGAS-interferon gene stimulating factor (STING) signaling (Figure 1) [6,8]. Moreover, along with apoptosis, the activated BAK and BAX are further oligomerzed on the OMM to enlarge the BAK/BAX pore, thus facilitating the release of mtDNA [8]. Furthermore, mtDNA is also released into extracellular environment through BAK/BAX pores to mediate innate immune responses under other conditions of non-apoptotic stress, such as viral infection, liver injury, acute kidney injury, inflammation, and toxic compound exposure [9,10,11,12,13]. To be specific, the absolute and relative amounts of BAK and BAX determine the rate of growth of the BAK/BAX pore and the kinetics of mtDNA release [14]. The intact mtDNA is released through the BAK/BAX pore [15], while mtDNA fragments are released through mPTP [16].

### 2.2. mPTP

The mPTP is a pore complex which exists at the contact point of inner and outer membrane of mitochondria and has the property of reversible opening and closing. The normal low permeability opening allows molecules with molecular weight less than 1500KDa to pass through, while abnormal high permeability opening results in decreased mitochondrial membrane potential, facilitating the release of mitochondrial DAMPs. The main components of mPTP include voltage-dependent anion channel (VDAC), adenine nucleotide translocator (ANT) and cyclophilin D (CypD). VDAC is located on the OMM and plays a crucial role in the formation of mPTP, in turn, the opening of mPTP also induces VDAC oligomerization [17,18]. In addition, VDAC is necessary for BAK/BAX-mediated OMM permeability and apoptosis [6]. The opening and closing of mPTP are controlled by multiple factors, such as oxidative stress, Ca^2+^ level, and cyclosporine A. In particular, ROS is a strong inducer of mPTP opening, and excessive ROS accumulation leads to continuous opening of mPTP which further leads to mitochondrial swelling and rupture.

Ca^2+^ enters mitochondria via mitochondrial calcium uniporter (MCU), inducing mPTP opening and VDAC oligomerization under non-apoptotic stresses such as radiation, exposure to toxic substances, viral infection, and gene mutation [18,19]. At the same time, the intact ox-mtDNA that formed in the mitochondria is cleaved into short fragments (500–650 bp) by Flap Endonuclease 1 (FEN1), which are then released into cytosol through mPTP to activate the NLRP3 inflammasome and cGAS-STING pathway [18,20] (Figure 1). Furthermore, once in the intermembrane space, mtDNA binds to the N terminus of VDAC via critical lysine and arginine residues and promotes the oligomerization of VDAC and the release of mtDNA, deficiency of VDAC oligomerization mitigates innate immune responses [16], suggesting that VDAC is required for the release of mtDNA into cytosol under non-apoptotic stress. However, in macrophages that treated with inflammasome agonists, the mPTP opening and VDAC oligomerization can occur independently of mtDNA binding and ROS oxidation [18,19]. As a result, how VDAC is involved in mPTP formation and the precise mechanisms by which mtDNA is released through mPTP require further elucidation.

### 2.3. GSDMD Pore

The GSDMD pore is composed of an active amino terminal GSDMD-NT and a self-inhibiting carboxyl terminal GSDMD-CT. Gasdermin is an effector molecule of pyroptosis, which includes classical and non-classical pyroptosis. The classical way refers to the recognition of PAMPs or DAMPs through PRRs, leading to the activation and assembly of inflammasome and caspase-1 [21]. In the non-classical way, lipopolysaccharide (LPS) induces pyroptosis by activating mouse caspase-11 and its human homology caspase-4/5 [22,23]. The GSDMD-NT fragments that formed after activation of caspase-1/4/5/11 are transferred to the cell membrane or mitochondrial membrane, or bind to acidic lipids and are then oligomerized and inserted into the membrane to form the GSDMD pore [21,24]. Subsequently, cell contents such as inflammatory cytokines interleukin-1β (IL-1β) and IL-18 are released from the GSDMD pore, and the osmotic pressure within the cells is destroyed at the same time, causing cell swelling and rupture, and finally inducing cell pyroptosis [21,25]. In addition to caspase-1/4/5/11, there are other pathways to activate GSDMD, such as caspase-8, which is also shown to cut GSDMD to induce pyroptosis [26,27,28].

Apart from the inflammatory cytokines, mtDNA is also released into cytosol through GSDMD pore (Figure 1). For example, the deletion of X-box binding protein 1 (XBP1) in hepatocytes results in the production of mitochondrial ROS (mtROS) by impairing mitophagy, thus activating the NLRP3-caspase-1-GSDMD signaling pathway to form the GSDMD pore. The mtDNA in hepatocytes is released to extracellular environment through the GSDMD pore and is then engulfed by macrophages, thereby activating the cGAS-STING signaling pathway in macrophage [29]. Similarly, mtDNA is released into cytosol through the GSDMD pore during non-classical pyroptosis to activate the cGAS-STING signaling pathway in endothelial cells [30,31,32]. Moreover, ox-mtDNA and GSDMD interact directly in neutrophils from patients with systemic lupus erythematosus, thereby promoting GSDMD-NT oligomerization, pore formation, and cell death [33]. Taken together, the GSDMD pore that formed in response to apoptotic stimulations not only facilitates the release of inflammatory cytokines into extracellular environment to trigger apoptosis, but also allows the release of mtDNA, thereby aggravating the innate immune response.

However, it is worth noting that there are still many open questions concerning the mechanisms that mediate the release of mtDNA. For example, whether mtDNA can be released from the above two or three pores at the same time, are there interactions between these pores, and if there is any other pore mediating the release of mtDNA. Last but not least, little is known about the upstream mechanisms mediating mtDNA release to date. Our previous study found that K^+^ efflux in macrophages led to mitochondrial damage and extracellular leakage of mtDNA, which then evoked the NLRP3 inflammasome [34], but it is not yet known whether the mtDNA release that induced by K^+^ efflux is accomplished through these pores.

## 3. MtDNA-Driven Innate Immune Signaling

**Figure 2:**
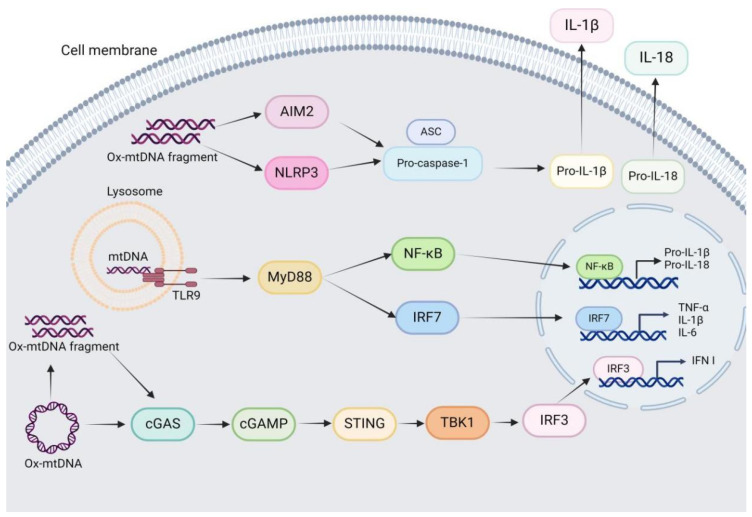
mtDNA-driven innate immune signaling. The mtDNA that released into cytosol binds to immune receptors cGAS, TLR9, NLRP3, or AIM2 to trigger innate immune responses. TLR9 translocates to the endosome membrane where it recognizes the unmethylated CpG in mtDNA and subsequently activates MyD88, promoting the nucleus translocation of NF-κB and IRF7 to upregulate the expression of pro-inflammatory cytokines and interferon, respectively. In addition, the cytosolic mtDNA can be recognized by cGAS, thus promoting the production of cGAMP which binds to and activates STING, leading to the phosphorylation of TBK1, thereby inducing the nucleus translocation of IRF3 to enhance the expression of IFN-I and ISGs to elevate innate antiviral immune response. Besides, the cytosolic NLRP3 recognizes and binds to ox-mtDNA to form the NLRP3 inflammasome with ASC and pro-caspase-1, inducing the maturation of pro-caspase-1 which cuts pro-IL-1β and pro-IL-18 to produce mature forms of IL-1β and IL-18. Similarly, the mtDNA is also recognized by AIM2 to form the AIM2 inflammasome with ASC and pro-caspase-1.

As stated before, mtDNA is released from mitochondria into cytosol, extracellular environment or circulating blood through BAK/BAX pore, mPTP and GSDMD pore under pathological conditions, and is then recognized by various PRRs including TLR9, cGAS and NLRP3 inflammasome to trigger innate inflammatory response and antiviral response (Figure 2).

### 3.1. mtDNA-TLR9 Signaling

TLRs are a family of ten innate immune sensors that are primarily expressed within innate immune cells and trafficked to endosomes or the plasma membrane upon stimulation. TLR9 recognizes DNA within the endosome that is enriched for hypomethylated CpG motifs in mtDNA [15,35]. TLR9 is localized in the cytoplasm or endoplasmic reticulum, while it translocates to the endosome membrane and binds to its ligand upon recognition of hypomethylated CpG motifs in DNA, which then activates the myeloid differentiation primary response gene 88 (MyD88), inducing the translocation of nuclear factor kappa-B (NF-κB) and interferon regulatory factor 7 (IRF7) into nucleus to enhance the expression of pro-inflammatory cytokines and type I interferon (IFN-I) respectively [15,36,37,38].

A large body of literatures support mtDNA as an endogenous TLR9 agonist (Figure 2). In 2010, Hauser’s lab first reported that the CpG sequence of mtDNA could activate TLR9, which in turn activated the neutrophil mitogen-activated protein kinase (MAPK) signaling pathway [39,40]. Subsequently, the binding of mtDNA and TLR9 is observed in other cell types, such as plasma dendritic cells, B cells, and NK cells [41]. Furthermore, the hypomethylated CpG in mtDNA binds to the N-terminal C-type leucine-rich repeats of TLR9 [42].Consistently, circulating mtDNA has been implicated in TLR9-dependent inflammatory pathology of diverse diseases such as rheumatoid arthritis, atherosclerosis, hypertension, acute liver injury and non-alcoholic steatohepatitis [43], suggesting that targeting mtDNA may represent an potentially effective strategy to combat TLR9-dependent inflammatory diseases. However, whether mtDNA, ox-mtDNA, and mtDNA fragments can all bind to and activate TLR9, and whether the binding ability differs between different forms of mtDNA, are elusive.

### 3.2. mtDNA-cGAS–STING Signaling

The cGAS is a major cellular DNA sensor, and recognizes exogenous DNA from pathogens and endogenous double-stranded DNA, leading to the activation of cGAS and the generation of cyclic guanosine monophosphate-adenosine monophosphate (cGAMP). STING is an endoplasmic reticulum membrane-resident protein that binds to cGAMP, after which it translocates to the Golgi, where it activates TANK-binding kinase 1 (TBK1). TBK1, in turn, phosphorylates the transcription factor interferon response factor 3 (IRF3), allowing it to translocate into the nucleus and promote the expression of IFN-I and other interferon-stimulated genes (ISGs) to trigger innate antiviral responses [6,15,44].

In recent years, mtDNA has been identified as one of the ligands of cGAS, and the cytosolic mtDNA can activate cGAS-STING signaling (Figure 2). For example, TFAM knockout [45], mitochondrial nuclease Endog deficiency [16], mtDNA instability [46], DNA topoisomerase I mitochondrial gene silencing [47], vacuolar protein sorting 13 homolog C mutation [48], and benzalkonium chloride treatment [20] all induce the release of mtDNA into cytosol through mPTP, and subsequently activate the cGAS-STING pathway to promote the generation of interferon to enhance the innate antiviral immunity. Furthermore, the mtROS production is increased which then oxidizes mtDNA upon mitochondrial damage, the intact ox-mtDNA is cut by FEN1 into short fragments of 500–650 bp. Meanwhile, Ca^2+^ enters the mitochondria through MCU to induce the opening of mPTP and VDAC oligomerization, allowing the release of short ox-mtDNA fragments into cytosol where they bind to cGAS and NLRP3, thereby activating the cGAS–STING signaling and NLRP3 inflammasome [18]. However, different from NLRP3 inflammasome, there is no significant difference between the binding abilities of cGAS to ox-mtDNA fragments and other double-stranded DNA [49,50,51]. In addition, benzalkonium chloride treatment triggers the release of mtDNA, rather than ox-mtDNA, into cytosol, thus activating the cGAS-STING pathway [20]. These discrepancies across studies may result from the different research object, measurement methods, and so on. Additionally, it is essential to acknowledge that current published research on mtDNA-cGAS-STING signaling is limited, additional research is needed to clarify the role of mtDNA in cGAS-STING under different pathological circumstances.

### 3.3. mtDNA-NLRP3 Inflammasome Signaling

NLRP3 is a member of intracellular PRRs, consisting of N-terminal pyrin domain (PYD), the nucleotide-binding oligerization domain (NBD/NACHT) and C-terminal leucine-rich repeat domains (LRR) which are responsible for ligand recognition. The NLRP3 inflammasome, the most widely studied inflammasome, is composed of NLRP3, apoptosis speck-like protein containing a caspase recruitment domain (ASC) and precursor caspase-1 (pro-caspase-1). ASC is the adapter of NLRP3, and composed of the N-terminal PYD domain and the C-terminal caspase recruitment domain (CARD) [52]. Pro-caspase-1 is the effector of NLRP3, consisting of the N-terminal CARD domain, the catalytic domain (P20), and the C-terminal catalytic subunit (P10) [53]. The activation of NLRP3 inflammasome involves two steps: “priming” and “activation”. In the priming step, PRRs such as TLRs, bind to corresponding ligands, inducing the nuclear translocation of NF-κB to promote the transcriptional expression of NLRP3, IL-1β, and IL-18. In the activation step, NLRP3 is oligomerized in response to various endogenous and exogenous stimulations such as microbial composition, crystal, particulate matter, and ATP. The oligomerized NLRP3 binds to the PYD domain of ASC through its PYD domain to activate ASC, the activated ASC then interacts with the CARD domain of pro-caspase-1 through its own CARD domain, inducing the maturation of pro-caspase-1 which further cuts pro-IL-1β and pro-IL-18 to generate active IL-1β and IL-18 [1] (Figure 2). Simultaneously, the activated caspase-1 can cut GSDMD to form the GSDMD pore, thereby secreting IL-1β and IL-18 to extracellular environment.

As has already been pointed out, the “activation” step of NLRP3 inflammasome is induced by diverse endogenous and exogenous agonists, however, these agonists are structurally and functionally different, indicating that these agonists may activate NLRP3 inflammasome through common signaling. Currently, the well-established mechanisms for NLRP3 inflammasome activation include intracellular ion flow, mitochondrial damage, Golgi disassembly, lysosomal disruption and metabolic disorder [54,55]. In 2011, Nakahira et al. found for the first time that knockout of mitochondrial autophagy related genes led to the accumulation of damaged mitochondria, and then the mtDNA was released into cytosol to activate NLRP3 inflammasome. On the contrary, the NLRP3 inflammasome activity was significantly impaired upon mtDNA deletion [56]. Furthermore, as shown above, the mtROS production is increased which then oxidizes mtDNA upon mitochondrial damage, the intact ox-mtDNA is cut by FEN1 into short fragments of 500–650 bp. Meanwhile, Ca^2+^ enters the mitochondria through MCU to induce the opening of mPTP and VDAC oligomerization, allowing the release of short ox-mtDNA fragments into cytosol where they bind to NLRP3, thereby activating the NLRP3 inflammasome [18,57,58] (Figure 2). In turn, the NLRP3 inflammasome is required for the translocation of mtDNA to the cytosol in response to LPS and ATP stimulation [56], suggesting a negative feedback loop between mtDNA and the NLRP3 inflammasome. In addition, the released cytosolic mtDNA can also activate other inflammasome other than NLRP3 inflammasome (Figure 2). For example, cholesterol overload in macrophages, FFA treatment of HepG2 cells, and perfluoroalkyl treatment of macrophages all lead to the formation of BAX/BAK pore, resulting in the release of mtDNA into cytosol to further activate the Absent in melanoma 2 (AIM2) inflammasome [13,59,60].

Taken together, the mtDNA that released into cytosol or extracellular environment in response to pathologic conditions is an endogenous DAMP that can be recognized by innate immune receptors, thereby triggering the innate immune response. Moreover, activation of the innate immune signalings may facilitate the leakage of mtDNA release which further aggravates innate immune responses. It should be noted that aberrant innate immune responses are being implicated in a large number of pathologies, including autoimmune diseases, metabolic syndrome, neurodegeneration and cancer. However, although the role of mtDNA in innate immune responses has gained great attention in the past two decades, the clinical evidence is relatively insufficient. Hence, more clinical trials are required to confirm those changes in patients, to confirm whether the released mtDNA can be used as a biomarker for disease prediction and diagnosis, and if targeting mtDNA holds promise to combat these diseases.

## 4. Effects of Exercise on mtDNA

Exercise has long been regarded as an effective intervention for innate immune response. Chronic low- and moderate-intensity exercise are thought to improve the immune system, while high-intensity exercise impairs it. However, most of the current studies focus on the detection of immune function in the condition of exercise, the underlying mechanism by which exercise exerts beneficial effects on immune responses remains poorly understood. Notably, mtDNA has emerged as a highly acknowledged DAMP in innate immune responses in the past two decades. However, the potential role and molecular mechanism of mtDNA in exercise-mediated innate immune response have been rarely reported, and a systematic review is lacking. In view of this, PubMed is used as the data resource for articles with the following keywords: (exercise (title) or training (title) or physical activity (title)) and (mitochondrial DNA (title) or mtDNA (title)). A total of 33 articles are included, and these articles are classified as endurance training (15), resistance training (1), endurance training combined with resistance training (2), high-intensity interval exercise (HIIT) (1), HIIT combined with continuous moderate-intensity exercise (1), acute exercise (9) and specialized sports (4) according to the type of exercise. The levels of mtDNA in circulating blood, cytosol, skeletal muscle and other tissues are determined in these literatures. Among them, the increase of mtDNA content in circulating blood and cytosol represents mitochondrial dysfunction which results in mtDNA leakage from mitochondria into cytosol or extracellular environment. On the contrary, the increase of mtDNA copy number in skeletal muscle and other tissues implicates the enhancement of mitochondrial function.

As shown in Table 1, endurance training is performed in most of these studies, and treadmill exercise is the most commonly used form of exercise, followed by power cycling. As expected, moderate-intensity endurance exercise for 12 weeks decreases circulating free mtDNA (cf-mtDNA) in blood of subjects with non-irritable bowel syndrome (non-IBS) [61]. In line with this, 8-week endurance exercise reduces the cytosolic ox-mtDNA levels in hepatocytes of mice with nonalcoholic fatty liver disease (NAFLD), thereby inhibiting the overactivation of the NLRP3 inflammasome [62]. However, moderate-intensity endurance exercise for 4 months upregulates plasma cf-mtDNA levels in patients with moderate to severe chronic kidney disease (CKD), there is more cf-mtDNA in individuals with combined moderate-intensity endurance exercise and caloric restriction than exercise intervention group [63]. These discrepancies may be due to the different exercise volumes performed by participants, more clinical and animal investigations are merited to illustrate the effects of moderate-intensity endurance exercise with different type and volume on the release of mtDNA. On the other hand, moderate-intensity endurance exercise for 2 weeks increases mtDNA in peripheral blood mononuclear cells of healthy subjects [64], the young adults with moderate-intensity endurance exercise for more than 6 months have higher leucocyte mtDNA copy numbers than the non-exercising group [65], the mtDNA copy number in skeletal muscle of healthy young individuals is increased significantly after 12-week endurance training [66,67], while 12-week endurance training exhibits no significance effect on mtDNA copy number in skeletal muscle of middle-aged participants [68], indicating that chronic moderate-intensity endurance exercise promotes mtDNA transcription and replication, especially for young individuals, thereby increasing mitochondrial content and enhancing mitochondrial function. Similarly, animal studies show that chronic low-, moderate- and high-intensity endurance exercise enhance the mtDNA contents in most brain regions [69,70,71], soleus [70] and vascular endothelial cell [72], with no obvious alterations in liver [73] and gastrocnemius [74]. By contrast, continuous exhaustive forced swimming for 7 days results in mtDNA damage in heart tissue of mice [75]. Compared to endurance exercise, few studies by far have investigated the impact of resistance exercise on mtDNA. Weight loaded-ladder climbing for 8 weeks elevates mtDNA copy number in skeletal muscle of rats [76]. Moreover, concurrent endurance and resistance training from 17 weeks gestation until birth significantly upregulates the mtDNA copy number in placenta of pregnant women [77], while combined endurance and resistance training for 26 weeks exhibits no remarkable effect on mtDNA copy number in blood of sugar, hypertension, and physical activity cohorts [78], suggesting that chronic concurrent endurance and resistance exercise can promote mitochondrial biogenesis, but there is no significant improvement in mitochondrial dysfunction, more evidence is needed to verify the effects of concurrent exercise on mtDNA in the future. Apart from the traditional endurance and resistance exercises, HIIT has also been recognized as an effective intervention in recent years. HIIT for 12 weeks enhances mtDNA content in skeletal muscle of type 2 diabetes mellitus (T2DM) [79], but HIIT combined with moderate- to high-intensity continuous exercise for 6 weeks has no significant effect on mtDNA content in skeletal muscle of healthy sedentary men [80], suggesting that the effect of HIIT on mtDNA content in human skeletal muscle depends on the time and volume of exercise, and more than 6 weeks of HIIT intervention may significantly promote mtDNA transcription and replication in skeletal muscle. Except for chronic exercise, the influence of acute exercise on mtDNA is also discussed here. Moderate-intensity acute exercise decreases mtDNA levels in gastrocnemius of rats [81], and decreases the cf-mtDNA in blood [82]. Moreover, the increased cf-mtDNA induced by exhaustive exercise drops rapidly to baseline levels during recovery [83,84,85,86,87]. While Acute high-intensity exercise has no effect on cf-mtDNA in blood [88,89] suggesting that the influence of acute exercise on mtDNA depends largely on the exercise intensity, acute moderate-intensity exercise inhibits mtDNA leakage, while acute high-intensity exercise exacerbates mitochondrial dysfunction and induces mtDNA leakage. Last but not least, specialized sports display similar positive effects on mtDNA compared to chronic moderate-intensity endurance exercise. Accident-free divers have less cf-mtDNA than non-divers [90]. Consistently, mtDNA levels are lower in plasma of professional volleyball players (PVPs) than in nonathletes, and cf-mtDNA is decreased in the first session [91]. On the other hand, world-class track and field master athletes have higher mtDNA copy numbers in muscle than non-athletes [92]. Likewise, young elite football players have higher mtDNA copy numbers in lymphocytes and mononuclear cells compared to young untrained controls [93], indicating that football improves the number of mitochondria in immune cells, thus enhancing the immune function. But the mtDNA levels in lymphocytes of elderly handball players are significantly lower than the control group [93], This is because that mtDNA damage in elderly is aggravated with age which leads to compensatory increase in mtDNA number, while regular exercise reverses the increase of mtDNA number by mitigating the mtDNA damage in the elderly, the reduction of mtDNA level in the lymphocytes of elderly handball players therefore is a reflection of enhanced mitochondrial function. Collectively, these results indicate that the specialized exercise improves mitochondrial function via promoting mtDNA transcription and replication, thereby avoiding mitochondrial damage and mtDNA release, which in turn enhances immunological function.

In conclusion, the effects of exercise with different mode, intensity and volume on mtDNA in circulating blood, cytosol, and tissues have been detected. Nevertheless, it is worth noting that the current published research are limited, in particular, most of these studies merely focus on the effects of exercise on mtDNA leakage and mtDNA content, the underlying mechanism by which exercise exerts these effects on mtDNA leakage is rarely reported and remains largely unknown.

## 5. Effects of Exercise on mtDNA and mtDNA-Driven Innate Immune Response

Despite the well-established role of mtDNA in innate immune response, little is known about the effect of exercise on mtDNA-driven innate immune response. As noted above, chronic endurance exercise reduces the cytosolic ox-mtDNA levels in hepatocytes of mice with NAFLD, thereby inhibiting the overactivation of NLRP3 inflammasome [62]. In addition, the mtDNA level is increased while TNF-α, IL-1β and IL-6 are decreased in hypothalamus of obese mice with chronic endurance training [71]. These results indicate that chronic endurance exercise can improve mitochondrial function, inhibit the release of mtDNA, thereby mitigating the excessive inflammatory response. However, the increase of cf-mtDNA in patients with CKD that induced by chronic endurance exercise is positively correlated with the increase of anti-inflammatory factor IL-10, but no correlation with the inflammatory factors such as IL-6, IL-8, TNF-α and markers of oxidative stress [63]. The cf-mtDNA levels in blood of non-IBS controls are decreased in response to chronic endurance exercise intervention, but there is no significant alteration in anti-inflammatory factors IL-10 and pro-inflammatory factors IL-6, IL-8 and TNF-α [61]. These two studies together imply that chronic endurance exercise may elicit the anti-inflammatory effect by regulating mtDNA release and other signaling pathways simultaneously. In addition, the level of cf-mtDNA is decreased and IL-6 is increased after acute moderate-intensity exercise, this is attributed to the removal of cf-mtDNA by neutrophil extracellular traps and DNase during exercise. Meanwhile, acute exercise promotes the generation and secretion of IL-6 from skeletal muscle [82]. However, the cf-mtDNA levels, number of leucocytes and inflammatory markers are significantly increased during exercise compared to baseline and after 30 and 90 min of rest [87]. These two studies indicate that the effects of acute exercise on cf-mtDNA levels depend on exercise intensity, acute moderate-intensity exercise reduces mtDNA release to suppress excessive immune response, while acute high-intensity exercise exerts the opposite effect.

**Figure 3:**
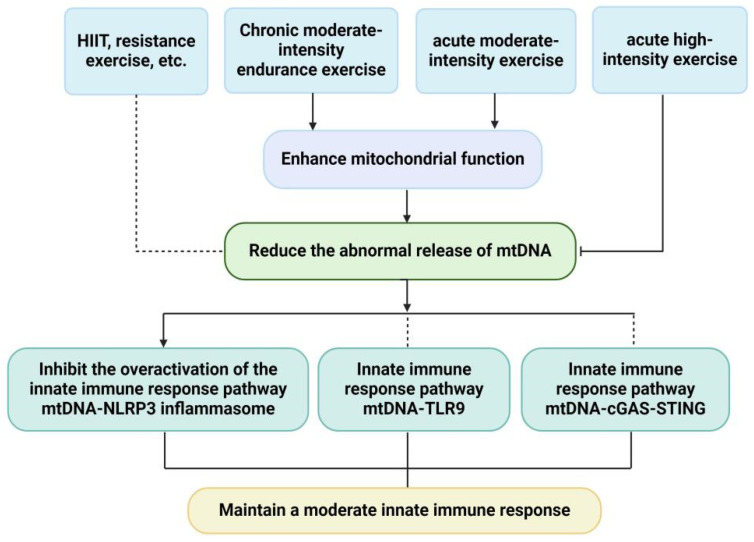
Effects of exercise training on mtDNA and mtDNA-driven innate immune. Chronic moderate-intensity endurance exercise enhances mitochondrial function, blunts the abnormal release of mtDNA, thereby inhibiting the overactivation of NLRP3 inflammasome. However, the effects of chronic moderate-intensity endurance exercise on other innate immune pathways such as mtDNA-TLR9 and mtDNA-cGAS-STING signaling remain unclear. In addition, the effects of HIIT and resistance exercise on mtDNA and mtDNA-driven innate immune response are ill-defined. At the same time, acute moderate-intensity exercise enhances mitochondrial function, thereby reducing the abnormal release of mtDNA, while acute high-intensity exercise hinders the transcription and replication of mtDNA and promotes the abnormal release of mtDNA, resulting in excessive innate immune response. The solid line is already validated, and the dotted line is to be verified.

To sum up, only a very small body of researches so far investigate the effect of exercise on mtDNA-mediated innate immune response, and there are many remaining questions. It is unclear whether moderate exercise can maintain appropriate innate antiviral and anti-inflammatory immune responses by directly inhibiting abnormal mtDNA leakage and the overactivation of mtDNA-TLR9 and mtDNA-cGAS signaling pathways. Except for chronic endurance exercise, whether other forms of exercise, such as resistance exercise and HIIT can attenuate excessive immune response by inhibiting the abnormal release of mtDNA, has not been reported (Figure 3). In view of the complex mechanism of exercise on mtDNA-driven innate immune response, additional research is merited to gain insight into the mechanism underlying the effects of exercise on mtDNA-driven innate immune responses.

## 6. Conclusions and Perspectives

The mtDNA, which is well known for its role as an important hereditary substance, is increasingly recognized as an agonist of the innate immune system during the past two decades. The mtDNA is released into cytosol, extracellular environment or circulating blood upon mitochondrial damage, where it is recognized by innate immune receptors, leading to excessive immune responses. Exercise has been considered to be an effective intervention strategy for innate immune response. Accordingly, exercise is likely to maintain the appropriate innate immune response through mediating mtDNA release. However, as a relatively nascent field, there are many remaining questions to date: (1) Most of the current studies mainly concerns about the innate immune response mediated by mtDNA, by contrast, the upstream molecules or signaling pathways regulating mtDNA leakage are largely unexplored. In particular, the underlying mechanism by which exercise affects mtDNA leakage is rarely reported; (2) Although the crucial role of abnormally released mtDNA in innate immune responses has been confirmed in a large number of animal and cell studies, it is short of compelling evidence that cf-mtDNA can be used as a clinical diagnostic biomarker for immune diseases and targeting mtDNA holds promising to combat immunopathies; In the future, the clinical significance of mtDNA in immune diseases needs to be verified. (3) Up to now, very few studies have been performed to illustrate the effects of exercise on mtDNA leakage and mtDNA-driven innate immune response, there is little direct evidence that regular endurance exercise, resistance exercise, concurrent endurance and resistance exercise, and HIIT can maintain the appropriate innate immune response by mediating mtDNA release; (4) Given the crucial role of mtDNA in innate immune responses, efforts to develop mtDNA-based drugs for treating immunopathies are very meaningful but limited to date. Moreover, combination of the mtDNA-based therapy with conventional exercise intervention is a promising therapeutic approach for immunopathies. (5) Lastly, despite the pivotal role of mtDNA in innate immune responses, little is known about the potential effect of mtDNA in adaptive immune response. Consequently, we are far from fully understanding the role of mtDNA in exercise-mediated innate immune responses, future work to address these concerns will have broad implications for a better understanding of the immune aetiology of human diseases and perhaps provide new avenues for therapeutic intervention to improve human health.

## Figures and Tables

**Table 1:** Effects of exercise on mtDNA.

Object	Exercise Type/Intensity/Time	Effects on mtDNA	Reference
Endurance Training			
Non-IBS Controls (n = 17); Patients with IBS (n = 26)	Walking, 60/75% of HR_max_, 5–10 km/h, 60 min/time, 3 times/week, 12 weeks	Exercise reduces cf-mtDNA in controls but not in patients with IBS	[61]
NAFLD mice	Treadmill training, 12 + 2 m/min/2 weeks, 1 h/day, 6 days/week, 8 weeks	Exercise decreases the cytosolic ox-mtDNA in hepatocytes of NAFLD mice	[62]
Patients with CKD (n = 99)	Treadmill, elliptical cross-trainer, NuStep cross-trainer, stationary recumbent bicycle, 60–80% VO_2max_, 30–45 min for 3 times/week for 4 months	Endurance exercise for 4 month increases plasma cf-mtDNA levels in patients with moderate to severe CKD	[63]
Healthy workers (n = 169); Healthy workers with exercise (n = 187)	Jogging for 30 min, 5 days a week for 2 weeks	Exercise increases mtDNA in peripheral blood mononuclear cells	[64]
Regularly exercising young adults (n = 44); non-exercising controls (n = 44)	The regular-exercising person is defined: a minimum of 300 min per month for more than 6 months	The regular-exercising group has higher leucocyte mtDNA copy numbers than the non-exercising group	[65]
Healthy elderly volunteers (3 women and 5 men)	Treadmill, stationary bicycles or outdoor walking, 4–6 sessions/week, 12 week. 30 min at 50–60% VO_2max_ for the first 4 weeks; 40 min at 50–60% VO_2max_ for next 4 weeks; ≥40 min at ~70% VO_2max_ for last 4 weeks	Exercise increases skeletal muscle mtDNA copy number	[66]
Healthy young (HY, n = 10); Healthy older (HO, n = 10); COPD (n = 20)	Cycling, 65% VO_2max_, 3 sessions of 30 min per week, 8 weeks	Exercise increases muscle mtDNA copy number in HY group, but not HO or COPD group	[67]
14 middle-aged participants (7 male/7 female)	Stationary bicycles, treadmill, or walking, 4–6 sessions/week, 30 min at 50–60% VO_2max_ for the first 4 weeks; 40 min at 50–60% VO_2max_ for next 4 weeks; ≥40 min at ~70% VO_2max_ for last 4 weeks	Exercise increases mtDNA content of skeletal muscle mildly without significance	[68]
Male ICR mice	Treadmill training, 25 m/min, 1 h/day, 6 days/week, and a 5% incline	Exercise increases mtDNA in most brain regions and soleus	[70]
Mice	Treadmill training, 8 m/min, 20 min for the first week; 12 m/min, 30 min for the second week; 4 m/min, 45 min for the third week; 16 m/min, 60 min for the fourth week; 18 m/min, 90 min for the fifth week	Exercise increases mtDNA content in hypothalamus	[71]
CAG_140_ knockin mice	Treadmill training, 8 ± 0.5 m/min for first day; 10 ± 1.5 m/min for 8 weeks; 20 ± 1.5 m/min, 40 min/time, 3 times/week, 12 weeks	Exercise elevates the mtDNA/nDNA ratio in brain	[69]
Mice	Voluntary wheel running, 1.8 km/h, 10 km/day, 7 weeks	Exercise enhances mtDNA in endothelial cells	[72]
Mice	Exhausting forced swimming, 1 h/day, 7 days	continuous exhaustive forced swimming for 7 days results in mtDNA damage in heart tissue of mice	[75]
PolG mutant mice	Voluntary wheel running, 10 months	Exercise has no impact on brain and liver mtDNA copy number	[73]
Apolipoprotein E knock-out mice with lower extremity artery disease	Treadmill training, 9 m/min for 3 min, with an increase of 2 m/min every 3 min until 19 m/min, 5 days/week; voluntary wheel running, 7 days/week; forced swimming, 60 min/day, 5 days/week; 4 weeks	Exercise has no obvious effect on hindlimb muscle mtDNA content	[74]
Resistance training			
SD rats (untrained, training, pre-training, re-training)	Weight loaded-ladder climbing, The amount of weight load was initially set at 50% of the body weight and gradually increased up to 300%. Each training session consisted of 3 sets of 5 climbing repetition, each rat was trained twice a day every third day for 8 weeks.	mtDNA copy numbers are significantly higher in re-trained muscles compared to the others	[76]
Concurrent endurance and resistance training			
Women (n = 47)	Concurrent endurance and resistance training, three 60-min sessions/week, from the 17th gestational week until birth	Exercise increases mtDNA copy number in placentas	[77]
Sugar, hypertension, and physical exercise cohorts (n = 105)	Endurance and resistance training for 45 min, 3 times/week, 6 month	Exercise has no significant effect on mtDNA copy number in blood	[78]
HIIT			
Healthy volunteer (n = 20); T2DM male patients (n = 30)	Treadmill training, 4 × 4 min intervals at 80–85% of HR_max_, with 3-min active recovery at 70% of HR_max_ between intervals, 40 min/time, 3 times/week, 12 weeks	Exercise enhances mtDNA content of skeletal muscle of T2DM patients	[79]
HIIT and moderate-high continuous exercise			
Healthy, sedentary male subjects (n = 10)	One-legged knee-extensor exercise, containing two HIIT and two moderate-high continuous exercise per week, 4 times/week, 6 weeks.	Exercise has no effect on mtDNA in trained leg	[80]
Acute exercise			
Male Zucker lean and Zucker obese rats	A single session swimming test, swam freely for first 30 min, and the next 30 min swimming was stimulated with the manual movement of water.	Acute exercise decreases mtDNA levels in gastrocnemius of lean and obese rats	[81]
Healthy moderately trained young men (n = 7)	Treadmill training, 60% VO_2max_, 90 min	Cf-mtDNA is declined when exercised for 54 min and immediately after exercise.	[82]
Healthy men (n = 11); T1DM patients (n = 14)	Treadmill run to exhaustion at 70%VO_2max_ at three consecutive days, separated by a 72 h resting period.	Each bout of exhaustive exercise increases cf-mtDNA.	[83]
Healthy controls (n = 11); T1DM men (n = 14)	Treadmill training, 1.5% incline, 70%VO_2max_ to exhaustion	The increase in cf-mtDNA concentration is significantly different between groups only in the second bout.	[86]
Young, healthy men (n = 20)	Exhaustive treadmill exercise, 15% incline, starting with a 5 min walking period at 1 m/s, increased by 0.2 m/s every 30 s afterwards until subjective exhaustion.	Circulating cf-mtDNA increases with peak levels at 15 min after exercise, and then rapidly drops to baseline levels.	[85]
Average-trained men (n = 11)	Three treadmill exercise tests to exhaustion at 70%VO_2max_ separated by 72 h of resting.	Cf-mtDNA rises significantly after the second and third bout of exercise, and decreases during recovery.	[84]
Healthy volunteers (n = 8)	Controlled ergo-spirometry cycle test, the resistance began at 30 W and 50 W for female and male, and increased by 10 W/min and 15 W/min respectively until exhaustion.	Cf-mtDNA significantly increases during exercise, compared to baseline values and after 30 and 90 min of rest	[87]
Healthy, physically active, non-smoking men (n = 5)	An incremental treadmill exercise test with a starting speed of 6 km/h and increased by 2 km/h every 3 min with 1.5% incline until exhaustion.	There is no difference in mitochondrial cf-DNA before and after exercise	[88]
Well-trained male athletes	Incremental treadmill exercise, 1% incline, the speed was increased by 2 km/h every 3 min until exhaustion.	Cf-mtDNA concentrations are not affected by exercise	[89]
Specialized sports			
Healthy nonathlete volunteers (n = 20); PVPs (n = 12)	Volleyball, 2 consecutive seasons (from fall to spring) from 2010 to 2012, 15 h per week.	mtDNA levels are lower in plasma of PVPs than in nonathletes, cf-mtDNA is decreased only in the first session, with no variations in the second session.	[91]
Non-divers (n = 22); accident-free divers (n = 8)	diving	Accident-free divers have less cf-mtDNA than non-divers.	[90]
Non-athlete controls (n = 14); World-class track and field master athletes (n = 15)	Track and field	World-class track and field master athletes have higher mtDNA copy numbers in muscle than non-athletes.	[92]
Young untrained controls (n = 30); young elite football players (n = 29)	football	Young elite football players have higher mtDNA copy numbers in lymphocytes and mononuclear cells compared to young untrained controls.	[93]
Elderly untrained controls (n = 35); elderly team handball players (n = 35)	Team handball	Elderly team handball players have lower mtDNA copy numbers in lymphocytes compared to elderly untrained controls.	[93]

IBS, irritable bowel syndrome; HR, heart rate; NAFLD, nonalcoholic fatty liver disease; CKD, chronic kidney disease; VO_2_, maximal aerobic capacity; COPD, chronic obstructive pulmonary disease; HIIT, high-intensity interval exercise; T2DM, type 2 diabetes mellitus; T1DM, type 1 diabetes mellitus; PVP, professional volleyball player.

## Abbreviations

PAMPs	pathogen-associated molecular patterns
DAMPs	damage-associated molecular patterns
PRRs	pattern recognition receptors
mtDNA	mitochondrial DNA
TFAM	mitochondrial Transcription Factor A
ROS	reactive oxygen species
ox-mtDNA	oxidized mtDNA
TLR9	toll-like receptor-9
cGAS	cyclic GMP-AMP synthase
NLRP3	nucleotide-binding oligomerization domain-like receptor family, pyrin domain-containing 3
BAK	Bcl-2-associated K protein
BAX	Bcl-2-associated X protein
mPTP	mitochondrial permeability transition pore
GSDMD	Gasdermin D
OMM	mitochondrial outer membrane
STING	cGAS-interferon gene stimulating factor
ANT	adenine nucleotide translocator
CypD	cyclophilin D
VDAC	voltage-dependent anion channel
MCU	mitochondrial calcium uniporter
FEN1	Flap Endonuclease 1
LPS	lipopolysaccharide
XBP1	X-box binding protein 1
NF-κB	nuclear factor kappa-B
MAPK	mitogen-activated protein kinase
cGAMP	cyclic guanosine monophosphate-adenosine monophosphate
TBK1	TANK-binding kinase 1
IRF3	interferon response factor 3
IFN-I	type I interferon
ISGs	interferon-stimulated genes
PYD	pyrin domain
NBD/NACHT	nucleotide-binding oligerization domain
LRR	leucine-rich repeat
ASC	apoptosis speck-like protein containing a caspase recruitment domain
pro-caspase-1	precursor caspase-1
CARD C	terminal caspase recruitment domain
IL-1β	interleukin-1β
mtROS	mitochondrial ROS
AIM2	Absent in melanoma 2
MyD88	myeloid differentiation primary response protein 88
IRF7	interferon regulatory factor 7
cf-mtDNA	circulating free mtDNA
non-IBS	non-irritable bowel syndrome
NAFLD	nonalcoholic fatty liver disease
CKD	chronic kidney disease
T2DM	type 2 diabetes mellitus
HIIT	high-intensity interval exercise
PVPs	professional volleyball players
